# Similarities and Differences in the Psychosocial Development of Children Placed in Different 24-h Settings

**DOI:** 10.1007/s10826-017-0955-6

**Published:** 2017-11-13

**Authors:** Harmke Leloux-Opmeer, Chris Kuiper, Hanna Swaab, Evert Scholte

**Affiliations:** 1 Horizon Youth Care and Special Education, Rotterdam, The Netherlands; 2 Leiden University, Leiden, The Netherlands

**Keywords:** Foster care, Family-style group care, Residential care, Short-term psychosocial development, Cohort study

## Abstract

Similarities and differences in the (short-term) psychosocial development of children in foster care, family-style group care, and residential care were investigated in a sample of 121 Dutch children (*M* age = 8.78 years; SD = 2.34 years; 47% female; 59% Caucasian) one year after their initial placement. Pretest and posttest measurements were carried out at the substitute caregivers using the CBCL. The results were examined at group level and case level. At group level, the findings showed no evidence for higher effectiveness in favor to the family-oriented settings (foster care, and family-style group care), as hypothesized. By contrast, some small differences were found between foster care and family-style group care, in favor of the latter. At individual level, a more or less equal number of children (18%) with a clinical pretest score on psychosocial functioning clinically significant improved (behavioral normalization). An important concern is that a number of children without clinical psychosocial problems at the time of admission clinically significant deteriorated (behavioral aberration) in psychosocial functioning (20%). This might indicate a poor match between the risks, needs and responsivity of the child on the one hand and the chosen intervention on the other. Future research on factors that (prior and during placement) positively as well as negatively affect the child’s psychosocial development is needed to further clarify this finding.

## Introduction

Out-of-home placement is considered to be a good alternative when in-home (support) services insufficiently provides in a safe parenting climate and positive development of the child (Pinto and Maia 2013; Vanschoonlandt et al. 2013). Out-of-home (24-h) care can be perceived as a continuum of services which vary in their intensiveness and restrictiveness, ranging from least restrictive types of care (e.g., kinship or non-kinship foster care) to family-based settings with paid caregivers (family-style group care) to placement in a residential setting (Huefner et al. 2010; Washington State Department of Social and Health Services: Children’s Administration 2014).

In foster care, a child is placed with relatives (kinship foster care) or with a licensed foster family, mostly due to concerns for its safety. In case of short-term foster care, the child stays temporarily with a foster family, while the biological parents are supported to improve their family circumstances in preparation for reunification (Strijker et al. 2008). When reunification is no option, a foster family provides a stable alternate rearing situation in a family setting until the child reaches the age of 18 (long-term foster care) (Strijker et al. 2008). In contrast to the foster care process in the United States, adopting a foster child is very unusual in the Netherlands and other European countries (Holtan et al. 2013).

Family-style group care can be perceived as an intermediate type of care between foster care and residential care (Barth 2002; Huefner et al. 2010). It is commonly used for children who are in need of professional supervision in a family-based setting (De Baat and Berg-le Clercq 2013). Many synonyms are in use for this type of care (e.g., teaching family homes, family type homes, SOS children’s villages, socio-pedagogical homes) (Grietens et al. 2015; Harder et al. 2013). A typical family-style group home (mostly situated in a neighborhood), is where a group of six to eight children reside and receive daily professional supervision from group home parents (mainly a married couple), who are mostly pedagogically trained and live at the setting (Grietens et al. 2015; Ringle et al. 2010).

The term “residential care” reflects a continuum of 24-h services that vary from open residential to secure residential to inpatient psychiatric care (Barth 2002; Grietens et al. 2015). Residential settings vary in their size, target group (e.g., delinquents, disabled children, children with mental health disorders), and in the therapeutic components available, and serve children with specialized treatment needs (Chor et al. 2012; Grietens et al. 2015). Two essential differences between residential care and family-based settings such as foster and family-style group care can be highlighted. First of all, in residential care children are supervised by 24-h shift staff who are not residents of the home (Berrick et al. 1993; Butler and McPherson 2007). Additionally, residential treatment has an integrated treatment team in a therapeutic milieu at its disposal, to provide a consistent, integrated and extended treatment that a family setting can hardly offer by the strain or duration of distress that inevitably arises (Butler and McPherson 2007). However, fundamental purpose of (all types of 24-h) out-of-home care services is to provide for the child’s safety and to promote positive child development, though in different ways (Adoption and Safe Families Act of 1997, 1997).

There are no evidence-based guidelines regarding which type of 24-h out-of-home care is the most suitable for a child undergoing whichever circumstances that require out-of-home placement (Courtney 1998; James et al. 2012). The current policy is that a “least restrictive” and family-oriented setting is preferred, such as foster care or family-style group care (Harder et al. 2013; United Nations 2009, December 18). Opinions vary regarding the added value of residential care (Grietens et al. 2015; Strickler et al. 2016). Among the known disadvantages of residential care are its cost (Grietens et al. 2015; James 2011), and its controversial effectiveness (Grietens et al. 2015; Knorth et al. 2008; Strijbosch et al. 2015; van IJzendoorn et al. 2011). Nevertheless, residential care is currently an integral part of the care continuum (James et al. 2012; López and del Valle 2015; Preyde et al. 2011). In addition, various published studies suggest that residential care is suitable and effective for children with certain, often severe, risks and needs (Chor et al. 2012; Conn et al. 2015; De Swart et al. 2012; Grietens et al. 2015). Moreover, the UN Guidelines for the Alternative Care of Children (henceforth “UN guidelines”) state that residential care can be preferable if it is necessary and constructive in the interest of the child (United Nations 2009, December 18).

The discussion about the added value of residential care within the continuum of care in case of out-of-home placement mirrors two underlying themes. The first is the difference in how the “least restrictive” policy is interpreted. Currently, care allocation appears to be based on a multi-stage procedure which initially starts by providing a least restrictive type of care (usually foster care), which then has to prove to be ineffective before more restrictive types of care are implemented. This method, however, implies that a well-informed referral decision for the type of care which would be most responsive to the child’s specific presenting problems plays a secondary role in the care allocation (Grietens et al. 2015; Sunseri 2005). For a certain group (usually children with severe problems) this method results in a long history in social services, involving several placements and replacements which then reduces the chance of achieving favorable outcomes (James et al. 2012; Oosterman et al. 2007). Moreover, care allocation is also affected by other factors than the child’s clinical needs such as resource availability (Broeders et al. 2015), or local referral policy (Huefner et al. 2010), due to the lack of an evidence-based assessment tool to support the decision-making process (Chor et al. 2012).

The second underlying theme reflects the discussion on the usefulness of residential care with regard to the problems this sector has in demonstrating its effectiveness (Grietens et al. 2015). First of all, comparisons are hampered by the use of the term “residential care” as a collective name for all types of 24-h care in a service-providing institute. These facilities vary in their size, in reason for placement (crisis, care, cure), in location (in or out of the community), and in their therapeutic components (Grietens et al. 2015; James et al. 2012). In addition, the comparison of effectiveness is confounded by the differences in the characteristics of the target groups at admission (i.e., age, degree of behavioral problems and care history) between children in foster care, family-style group care and residential care (Leloux-Opmeer et al. 2017; Conn et al. 2015; Den Dunnen et al. 2012). These differences in treatment contexts and the differences in risks, needs and responsivity at admission, mean that the comparability of the outcomes of the existing types of care is limited (James et al. 2012; Preyde et al. 2011). This is why different researchers propose that a more realistic depiction of treatment effectiveness would be acquired if the outcomes in the different types of care were compared with the specific baseline situations with which the children and their families initially entered care (Conn et al. 2015; McCrae et al. 2010).

The aim of this study is to answer the question how the type and severity of psychosocial functioning at the time of admission affect the degree of (short-term) psychosocial development in different types of out-of-home care. To this end, similarities and differences in the psychosocial development of the out-of-home placed children were investigated at group level as well as at individual level during the first year (with a minimum of 3 months) after initially being placed in foster care, family-style group care and open residential care. First, we expect that foster children and children in family-style group care will experience a more favorable psychosocial development than children placed in residential care. Second, we hypothesize that children with severe psychosocial problems at admission develop less favorably at group level as well as at case level than children who do not have significant problems in this area. Additionally, we expect this prediction to be most clearly reflected in foster care.

## Method

### Participants

**Fig. 1:**
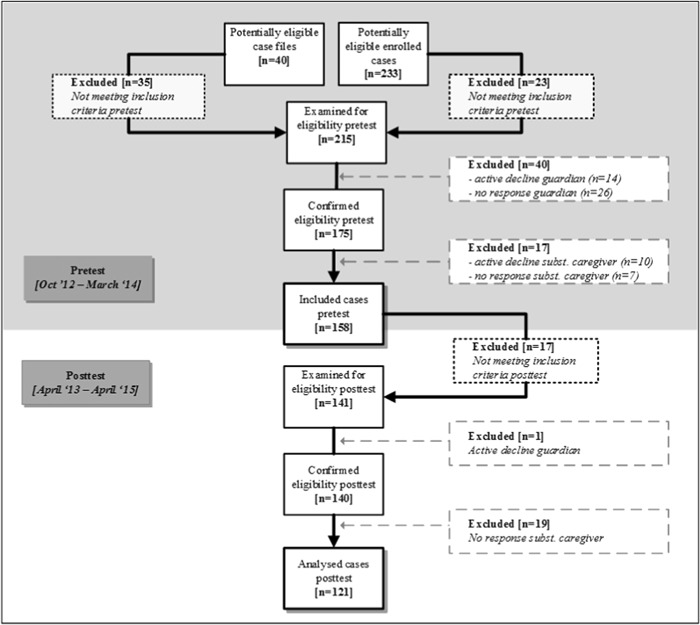
Flowchart showing the inclusion process for cases with a CBCL pre and posttest from substitute caregivers

The study was part of a larger cross-sectional cohort study with a broad set of instruments and informants. The study population consisted of Dutch out-of-home placed primary-school children (aged 4–12) in foster care (kinship or non-kinship), family-style group care and open residential care. In this particular study only cases from who a Child Behavior Checklist (CBCL) pretest of the substitute caregiver was available, were included. The inclusion process is represented in the flowchart in Fig. 1. Of the 158 cases who were included in the first, cross-sectional study, 17 cases were excluded because they did not meet the inclusion criteria for the posttest, mainly due to premature departure (*n* = 11). Of the 141 cases examined for eligibility, a posttest was completed by a substitute caregiver in 121 cases, with a mean response rate of 86% (foster parents 73%, family-style group parents 74%, group care workers 95%). The response rate was calculated by dividing the number of included respondents by the number of respondents examined for eligibility for the posttest (Morton et al. 2006; Sitzia and Wood 1998; The American Association for Public Opinion Research (AAPOR) 2011). The response rate is comparable with the median participation rate of 80% in cohort studies (Morton et al. 2006) and lies above the mean response of 61% of written questionnaires as reported by Cummings et al. (2001).

More important than the percentage of the response rate was to establish if the response group in the cohort study (*n* = 121) was representative for the eligible cases. Therefore, we compared the most important core pretest variables of this group with those of the group that was excluded because of non-eligibility or non-response (*n* = 37) (Galea and Tracy 2007; Werner et al. 2007). We found no significant differences, and effect sizes were negligible between the inclusion group and the exclusion group with regard to gender, child protective service custody, ethnicity, and socio-economic status (using Fisher’s exact tests). The same was true with regard to age at admission, degree of behavioral problems, degree of fundamental detachment, total care history, and total number of (re)placements (using two-tailed independent T-tests).

One third of the participating foster families was a kinship family. The mean number of children in the foster homes was 3.6 (SD = 1.3), with a mean number of 1.9 (SD = 1.3) biological children. Seventeen percent of the foster children received therapy or medication supplementary to the placement. Additionally, 13% went to special education. The participating foster parents, foster children and biological parents were counseled by foster care workers and a behavioral scientist. The participating family-style group homes accommodated an average of six (SD = 2.0) children, with a mean number of 1.4 (SD = 1.3) biological children. The group home parents received supervision from group home workers and a behavioral scientist, and they maintained contact with the biological parents. One in three children received therapy or medication, and 70% received special education. The included children in open residential 24-h care were placed in a structured living group (8–10 children), guided by group care workers and a behavioral scientist. In addition to group therapy, 48% received individual therapy or medication, and all children went to an incorporated school for special education.

### Procedure

Substitute caregivers were recruited from Horizon; a large Dutch organization for youth care and special education. The family-style group parents in the cohort study were paid employees or independent entrepreneurs. The subgroup was supplemented by two third with children from independent family-style group homes associated with other, comparable Dutch youth care organizations. In addition, we partially complemented the subgroup (14%) using retrospective record analysis of primary-school children who went through intake for a family-style group home of Horizon in 2011 or the first 9 months of 2012.

The data collection took place between April 2013 and April 2015. It comprised a posttest a minimum of 3 months and a maximum of one year after the pretest. The following were the inclusion criteria: (1) participation would not harm the treatment alliance with the parents, (2) a CBCL pretest of the substitute caregiver was available against which the posttest could be compared, and (3) there was a minimum of 3 months between the pretest and posttest. The exclusion criteria involved: (1) children in a crisis placement or placement in secure residential youth care, and (2) adopted children or children with profound intellectual disabilities (IQ < 70).

The cohort study was not subject to the Medical Research Involving Human Subjects Act (WMO) and was therefore not put before a medical ethics review committee. The study procedure satisfied the Netherlands Code of Conduct for Academic Practice (Association of Universities in the Netherlands 2014): (1) written consent was requested from the guardians before participation in the cohort study, (2) participants could withdraw from the study without explanation, and (3) file numbers were distorted to guarantee the participants’ anonymity.

### Measures

To determine the psychosocial development of the out-of-home placed children, we used two assessments forms of the Achenbach System of Empirically Based Assessment (ASEBA), specifically the *Child behavior checklist (CBCL)/1.5-5 and CBCL/4-18*. The Dutch versions of these two checklists were completed by substitute caregivers at the time of the pretest and posttest (Achenbach and Rescorla 2001; Verhulst et al. 1996). The CBCL/1.5-5 and CBCL/4-18 ask informants to use a three-point scale (where 0 = not true, 1 = sometimes true, and 2 = very true) to respectively assess 99 and 120 items relating to behavioral and emotional problems. The summary scale *T* scores of emotional (internalizing) problems (i.e., somatic complaints, withdrawn, and anxious/depressed behavior), behavioral (externalizing) problems (i.e., aggressive and delinquent behavior), and total psychosocial problems from both instruments were used in this study. Achenbach and Rescorla (2001) suggest to use *T* scores of 60 or above to discriminate between children with and without (borderline) psychosocial problems. The psychometric characteristics of the CBCL are regarded as satisfactory (Achenbach and Rescorla 2001).

A case file characteristics questionnaire (CCQ) was designed to chart file information systematically on demographic characteristics (e.g., age, gender, ethnicity), clinical characteristics (e.g., psychosocial problems, school or cognitive problems, child mental illness), family characteristics (e.g., family composition, clinical family problems), and care history characteristics (e.g., previous placements, child protective services) at admission. This 30-item questionnaire was completed by or under the supervision of a behavioral scientist. Most items of the CCQ were related to factual information, and all were categorized, and if possible, dichotomized (yes/no). For potentially ambiguous items which require some interpretation, a scoring protocol was available. The inter-observer reliability of the questionnaire was used to measure the intraclass correlation (ICC) of the CCQ (Cohen 1992). Five files were scored with the CCQ by two raters. Based on the guidelines by Landers (2015), a two-way mixed model was used, with absolute agreement as a criterion. The mean ICC (95% CI) was 0.66 (0.58, 0.72), which reflects a moderate inter-observer reliability (Shrout 1998).

### Data Analyses

SPSS 23 (Statistical Package for Social Sciences) was used for the data analysis. With Pearson’s correlation coefficient for continuous variables, and Student’s *t*-test for discrete variables we examined the effect of several important control variables which were distracted from literature data, on the outcome measure (difference scores on the CBCL). Assessment of the preconditions of all statistical tests were carried out before the analysis. Outliers were tested by calculating standardized z-scores. Responses with a *z*-score greater than 3.29 (*p* < .001, two-tailed test) were regarded as outliers (Cohen 1992). We handled outliers by taking the next highest score plus one unit (Cohen 1992).

We investigated the psychosocial development in out-of-home placed children during the first year of placement using 3 × 2 (Setting × Time) repeated measure (RM) ANOVAs with Sidak tests for multiple comparisons. This technique corrects for variation created by individual differences in performance (Cohen 1992). The size of the significant change was represented with partial eta squared. A value of .01 reflected a small effect, .06 for a medium-sized effect and .14 for a large effect (Cohen 1992).

We examined the influence of the psychosocial functioning at admission on the degree of development with 3 × 2 factorial ANOVAs followed by Sidak tests for multiple comparisons. Three new dependent variables were produced with difference scores (*T*_0_–*T*_1_) on psychosocial functioning, emotional functioning and behavioral functioning, in which a positive difference score reflected a positive development. One extreme outlier was found within the subgroup family-style group care for the difference score in behavioral problems (*T*_diff_ = 30). This score was replaced by the next high score plus one unit (*T*_diff_ = 23).

To gain insight into the individual development of children, we calculated the Reliable Change Index (RCI) (Jacobson and Truax 1991; Kline 2004). An RCI greater than 1.96 or smaller than −1.96 was regarded as statistically significant, corresponding to the significance value of *p < *0.05 (Jacobson and Truax 1991). A positive significant RCI indicated progress in individual development and a negative significant RCI indicated deterioration. A non-significant RCI meant there was no change. If the progress was accompanied by a transition of the clinical or subclinical to the normal domain of the CBCL, then a clinically significant improvement took place (Jacobson and Truax 1991). A shift from the normal range to the (sub)clinical range implied a clinically significant deterioration. Fisher’s exact test was used to investigate the association between the individually significant change and the offered type of care (Cohen 1992). The effect size was determined using Cramer’s V and could lie between 0 and 1 (Field 2009). A value of .10 represented a small effect, .30 for a medium-sized effect and a value equal to or greater than .50 for a large effect (Cohen 1992). In all cases a two-tailed test was used and *p*-values less than .05 were interpreted as statistically significant.

## Results

Of the analyzed cases for which both a pre and posttest on the CBCL were available, 30 came from foster care, 14 from family-style group care, and 77 from open residential care. See Table 1 for some of the demographics. Foster children were on average younger then children in family-style group and residential care at time of the admission. As regards gender, the subgroup children in family-style group care consisted of less boys compared to foster and residential care. Finally, the time between the pre and posttest (mean placement duration) was slightly higher for children in family-style group care than for foster children and children in residential care. In general, the placement was terminated at the time of the posttest for 22% of the included cases.

**Table 1:** Demographic characteristics at baseline for children in foster care, family-style group care and residential care

	FC	FGC	RC	Test	Effect size
	(*n*_*max*_ = *30*)	(*n*_*max*_ = *14)*	(*n*_*max*_ = *77)*		
Gender (% male)	43	29^a^	61^a^	*χ* ^2^(2,N = 121) = 6.47*	.23
Race (% Caucasian)	63	67	56	*χ* ^2^(2,N = 93) = 0.72^ns^	.09
Child protective services (%)	90	83	73	*χ* ^2^(2,N = 115) = 3.99^ns^	.19
SES (% low)	50	50	62	*χ* ^2^(2,N = 25) = 0.34^ns^	.12
Mean age at admission (yrs)	*M = *7.5, SD = 2.5^a,b^	*M = *9.7, SD = 2.1^a^	*M = *9.1, SD = 2.1^b^	*F*(2,120) = 6.97**	.11
Time between pre and posttest (mo)	*M* = 13.0, SD = 1.7^a^	*M* = 15.4, SD = 2.2^a,b^	*M* = 12.7, SD = 1.6^b^	*F*(2,120) = 15.05***	.20

*Note:* Means with the same superscript differ significantly

*FC* foster care, *FGC* family-style group care, *RC* residential care

*p < .05 (Chi-square test with Cramer’s V)

**p < .01; ***p < .001 (ANOVA with *η* ^2^)

Prior to the analyses, the influence of several core pretest variables on the outcome variables (difference scores on the CBCL) has been examined (see Table 2). Preliminary analyses showed medium sized significant Pearson’s correlations between the psychosocial functioning at admission and the degree of psychosocial development. Therefore, the influence of the baseline situation on the psychosocial development was subsequently investigated explicitly by adding it as a factor in the factorial analyses, in which children with a *T*-score of 60 or higher at admission were included in the “clinical group”. No significant associations were found between the development of psychosocial functioning and the discrete variables gender, socio-economic status, and ethnicity (measured with two-tailed independent Student’s t-tests).

**Table 2:** Descriptive information and correlations between pretest variables and degree of psychosocial development (difference scores on Total CBCL, Internalizing and Externalizing)

Variables	*M*	SD	DIFF Internalizing *r*	DIFF externalizing *r*	DIFF total problems *r*
Age at admission (*N* = 121)	8.78	2.34	0.09	−0.02	0.05
Number of previous placements (*N* = 110)	1.49	1.63	−0.01	0.09	−0.01
Length of care history (mo) (*N* = 63)	13.18	2.48	−0.03	0.12	0.08
Time between pre and posttest (*N* = 121)	13.08	1.91	0.11	0.07	0.09
Pretest internalizing problems (*N* = 121)	57.37	9.97	0.48***	0.14	0.32***
Pretest externalizing problems (*N* = 121)	58.55	11.79	0.17	0.44***	0.35***
Pretest total problems (*N* = 121)	59.27	10.43	0.26**	0.36***	0.42***

**p < .01; ***p < .001 (two tailed Pearson correlation *r*)

### Psychosocial Development During Placement

Table 3 shows the results of the repeated measures ANOVA for the psychosocial development during placement. With regard to the total psychosocial functioning, no significant main effects were found on time and setting. The Setting x Time interaction showed an almost significant effect, *F*(2,118) = 2.87, *p* = .06, *η²*_*p*_ = .05. This indicates that the psychosocial development of the children was related to the setting in which the child was placed. Post hoc analyses demonstrated that children differed in the degree of functioning in the three main types of care at the time of admission, *F*(2,118) = 3.30, *p* = .04, *η²*_*p*_ = .05, in which foster children showed significantly fewer psychosocial problems than children in family-style group care (*p* = .03). Post hoc analyses also demonstrated a trend of psychosocial problems increasing within foster care, Wilks’ Lambda = .97, *F*(1,118) = 3.65, *p* = .06, *η²*_*p*_ = .03. At the time of the posttest, these differences were no longer present.

**Table 3:** Progress in total psychosocial functioning, emotional, and behavioral development (CBCL) during a 1-year follow-up, arranged by setting (repeated measures ANOVA)

	Settings			Effects		
	FC (*n* = 30)	FGC (*n* = 14)	RC (*n* = 77)	Setting	Time	Setting x Time
	*M (SD)*	*M (SD)*	*M (SD)*	*F*	*F*	*F*
Total score CBCL				1.55	** <**1	2.87
Pretest *T*-score*	56.30(10.37)^a^	64.79(13.34)^a^	59.43(9.55)			
Posttest *T*-score	59.23(8.44)	61.50(15.14)	59.23(10.14)			
Internalizing CBCL				<1	<1	3.19*
Pretest *T*-score	55.57(9.01)	62.14(11.76)	57.21(9.84)			
Posttest *T*-score	58.03(8.01)	56.64(16.69)	57.99(9.68)			
Externalizing CBCL				<1	<1	1.55
Pretest *T*-score	54.67(11.85)	62.17(15.08)	59.31(10.83)			
Posttest *T*-score	57.30(10.46)	60.29(15.18)	60.08(10.70)			

*Note*: Means with the same superscript differ significantly

*FC* foster care, *FGC* family-style group care, *RC* residential care

*p < .05

There were no main effects on time and setting for emotional development. However, a significant interaction effect was found for Setting × Time, *F*(2,118) = 3.19, *p* = .04, *η*²_p_ = .05. Post hoc analyses showed that children in family-style group care developed more positively in the emotional domain than children in foster care or open residential care, Wilks’ Lambda = .96, *F*(1,118) = 4.31, *p* = .04, *η²*_*p*_ = .04.

There were neither main effects nor interaction effects for behavioral development. This suggests that the behavioral functioning and the behavioral development for children in all three types of out-of-home care were comparable at every moment during the first year of placement. The finding further suggests that the behavioral functioning in all three settings remained unchanged during the investigated placement duration.

### Influence of Psychosocial Functioning at Admission

Table 4 shows the results of the 3 × 2 (setting × severity at admission) factorial ANOVAs. As regards the total psychosocial development, there was a significant main effect of the severity of total problems at the time of admission on the degree of psychosocial development, *F*(1,120) = 5.04, *p* = .03, η²_p_ = .04. The mean T-score of the clinical group decreased by 2.5 (*SD* = 1.2) points, while the mean *T*-score of the non-clinical group increased by 1.9 (*SD* = 1.6) points. However, the mean posttest score of the non-clinical group was still within the normal range, M = 53.42, *SD* = 9.26. Furthermore, no significant main effect was found for setting (*p* = .12, *η²*_*p*_ = .04), and there was no significant interaction effect (*p* = .39, *η²*_*p*_ = .02).

**Table 4:** Factorial ANOVA with setting (FC, FGC, RC) and pretest score (clinical/non-clinical) as independent variables and difference scores on Total CBCL, Internalizing and Externalizing as the dependent variable (N = 121)

	*df*	*SS*	*MSE*	*F*	*p*	*η²*_*p*_
Total score CBCL						
Setting	2	279.01	139.51	2.15	.12	.04
Pretest score	1	327.31	327.31	5.04	*.03*	.04
Setting × pretest score	2	124.13	62.07	0.96	.39	.02
Residual	115	7472.64	64.98	–	–	–
Total	120	8742.98	–	–	–	–
Internalizing CBCL						
Setting	2	598.50	299.25	3.48	*.03*	.06
Pretest score	1	174.80	174.80	2.04	.16	.02
Setting × pretest score	2	443.55	221.78	2.58	.08	.04
Residual	115	9877.29	85.89	–	–	–
Total	120	12216.15	–	–	–	–
Externalizing CBCL						
Setting	2	58.67	29.34	0.41	.66	.01
Pretest score	1	276.64	276.64	3.86	.05	.03
Setting × pretest score	2	53.72	26.86	0.38	.69	.01
Residual	115	8231.66	71.58	–	–	–
Total	120	9267.17	–	–	–	–

Italic *p*-values are significant at *p* < .05

With regard to the emotional problems, there was no significant main effect for the severity of emotional problems at the time of admission on the emotional development (*p* = .16, *η²*_*p*_ = .02), and no significant interaction effect (*p* = .08, *η²*_*p*_ = .04). Nevertheless, a significant main effect was found for setting, *F*(2,120) = 3.48, *p* = .03, *η²*_*p*_ = .06. Post hoc analyses showed that children in family-style group care had, on average, developed significantly more positively than foster children (*p* = .03) when no difference was made between the clinical and non-clinical group. The mean T-score of the children in family-style group care reduced by 6.3 (*SD* = 2.7) points, while the mean T-score of foster children increased by 2.2 (*SD* = 1.7) points.

With reference to the behavioral development, there was an almost significant main effect for the severity of the behavioral problems at the time of admission, *F*(1,120) = 2.86, *p* = .05, *η²*_*p*_ = .03. The mean T-score of the clinical group on the externalizing problems scale reduced by 2.0 (*SD* = 1.3) points, while the mean T-score of the non-clinical group increased by 2.4 (*SD* = 1.8) points. There was no significant main effect for the factor setting (*p* = .66, *η²*_*p*_ = .01), and also no significant interaction effect between both factors (*p* = .69, *η²*_*p*_ = .01).

### Individual Development during Placement

Table 5 shows the percentages of children who experienced a statistically significant change during placement in terms of developmental progress, no change and developmental deterioration. In the analysis, a distinction has been made between the clinical and non-clinical group. In addition, the results of children in family-style group care were excluded from the non-clinical group, because of the low number of children with non-clinical pretest scores.

**Table 5:** RCI by psychosocial problems at admission (clinical/non-clinical) with Fisher’s exact test for association with setting

	%	%	%	Fisher’s exact
	Improvement	No change	Deterioration	Test with Cramers V
Clinical pretest score				
Total scale CBCL (*n* = 62)	35	50	15	*p* = .89, V = .10
Internalizing CBCL (*n* = 57)	35	47	18	*p* = .51, V = .16
Externalizing CBCL (*n* = 59)	39	27	34	*p* = .71, V = .14
Non-clinical pretest score^a^				
Total scale CBCL (*n* = 55)	15	40	45	*p* = .57, V = .17
Internalizing CBCL (*n* = 60)	22	32	47	*p* = .26, V = .22
Externalizing CBCL (*n* = 59)	20	17	63	*p* = .47, V = .16

^a^ Family-style group care is excluded because of the low number of children with non-clinical pretest scores

The table shows that 35–39% of the children in the clinical group had statistically significantly progressed in their psychosocial functioning, against 15–22% of the children in the non-clinical group. Additionally, 15–34% of children with clinical psychosocial problems at admission deteriorated any further, against 45–63% of children with no clinical problems at admission. When comparing developmental changes between the children in the three types of care, no statistically significant differences were found in type and degree of individual psychosocial development (Fisher’s *p* = .89), emotional development (Fisher’s *p* = .51), and behavioral development (Fisher’s *p* = .71) for children with clinical pretest scores. For the non-clinical group no statistically significant differences were found between foster and residentially placed children in individual psychosocial development (Fisher’s *p* = .57), emotional development (Fisher’s *p* = .26), and behavioral development (Fisher’s *p* = .47).

Developmental changes were considered to be clinically relevant when a statistically significant improvement is complemented by a normalization of the psychosocial functioning at the posttest. For the total psychosocial functioning, 11 of 62 (18%) children showed such a clinically relevant improvement. In addition, the emotional functioning of 14 of the 57 (25%) children normalized during placement. With regard to behavior problems, 11 of the 59 (19%) children showed a clinically relevant improvement. Due to the low numbers of children with a clinically relevant improvement, no comparison could be made between the three types of care.

Finally, individual children were classified as having a clinically significant deterioration in psychosocial functioning when this functioning is normal at the pretest but ends in the (sub)clinical domain at the posttest. This was the case in 11 of the 55 (20%) children with regard to the total psychosocial development. Furthermore, in 16 of the 60 (27%) children the emotional functioning shifted to the clinical domain during the first year after placement. With regard to the behavioral development, the posttest score of 16 of the 59 (27%) children shifted to the clinical domain. No comparison could be made between foster and residentially placed children because of the small number of children with a clinically significant deterioration.

## Discussion

Fundamental goal of all types of (24-h) out-of-home care is to provide for the child’s safety and to promote positive child development. To enlarge the knowledge of child development with regard to children in different types of out-of-home care, this study investigated similarities and differences in short-term psychosocial development of children placed out-of-home in foster care, family-style group care and residential care. Both analyses of changes at group level and changes at case level were explored. Moreover, the severity of the children’s psychosocial problems at the time of admission was taken into account to obtain an adequate impression of the effectiveness of the considered care modalities, as suggested by Connor et al. (2002), and Wilson et al. (2004).

Our findings did not confirm the first hypothesis that both family-oriented settings (i.e., foster care and family-style group care) will be more effective than open residential care, as proposed in the UN guidelines (United Nations 2009, December 18) and suggested by literature data (e.g., Courtney 1998; Harder et al. 2013). The psychosocial development between children in family-oriented settings and residential care were largely comparable at group level and individual level over a period of one year, according to substitute caregivers. Generally speaking, one third of the children experienced no developmental change, and one third respectively improved or deteriorated.

Even though the development in the three settings is broadly equal, some differences are noteworthy at a trend level. First of all, the average level of severity of psychosocial problems in foster children slightly increases during the first year of placement. This is in line with literature data (Lawrence et al. 2006; Vanderfaeillie et al. 2013). However, further analyses at case level showed that a decline in functioning mainly applies to foster children without clinical psychosocial problems at the time of admission. Even though the mean psychosocial functioning of foster children still fell within the normal range at posttest, the trend of increasing psychosocial problems might persist long term [as mentioned in the study by Lawrence et al. (2006)], which can ultimately increase the risk of a breakdown (Strijker et al. 2008; Van den Bergh and Weterings 2010; Vanschoonlandt et al. 2012).

Second, our findings indicated that children in family-style group care seem to have a more positive emotional development than foster or residentially placed children. Unfortunately, the data from our study could not provide a clear explanation for this, due to the relatively small number of children in family-style group care.

Finally, specifically with reference to care history, it is worth noting that preliminary analyses in this study did not confirm that a less favorable psychosocial development can be linked to unfavorable care history characteristics (i.e., number of placements, length of care history). A possible explanation for not finding a significant association between care history and psychosocial development might be that other factors such as the quality of care or the responsivity of the child (e.g., learnability) have mitigated the association. However, this should be explored in future research.

Our second hypothesis was that children with severe psychosocial problems at admission would develop less favorably than children without significant psychosocial problems. However, our data indicated the opposite. Results both at group and individual level demonstrated that children with clinical psychosocial problems at admission experienced a more positive development than children in the non-clinical group. Even though the psychosocial functioning of the children in the clinical group still fell, on average, within the clinical or subclinical range at time of the posttest; at individual level about one in five children (18%) had a clinically relevant improvement in global psychosocial functioning. This percentage falls within the range of earlier reported percentages of improvement (varying from 0 to 29%) for out-of-home placed children (Boyer et al. 2009; Vanderfaeillie et al. 2013). By contrast, one in five (20%) children in the non-clinical group experienced a clinically relevant deterioration in psychosocial functioning at individual level, although the results on group level fell (on average) still within the normal range at time of the posttest. This percentage of deterioration is also more or less consistent with literature data reporting percentages varying from 17 to 25% (Boyer et al. 2009; Vanderfaeillie et al. 2013).

Supplementary to the second hypothesis, we expected that children with clinical psychosocial problems would develop less favorably in foster care specifically, based on literature data showing that placement breakdowns (one-third) in foster care are mainly caused by the level of psychosocial problems at admission (Van den Bergh and Weterings 2010; Vanschoonlandt et al. 2012). However, in all three types of care the clinical group experienced a comparable positive development. This finding might indicate that the provided out-of-home care was attuned to the specific needs of the children and their responsiveness for the specific type of out-of-home care. On the other hand, an alternative explanation may be that regression to the mean has occurred, which is a common phenomenon in repeated measurements between groups (Barnett et al. 2005). Further experimental research is needed to clarify this.

Furthermore, with reference to the differences in development between the clinical and non-clinical groups the following two key issues are noteworthy. First, it is remarkable that a part of the out-of-home placed children did not have clinical psychosocial problems at the time of admission. Probably this especially concerns children who are placed out-of-home due to severe family circumstances (e.g., neglect, parental mental illness, incarceration). However, an out-of-home placement itself can be just as traumatic and can lead to behavioral problems (Bruskas 2008; Schneider and Phares 2005). This might explain the finding that particularly this specific group deteriorated with regard to psychosocial functioning during the first year of placement. More research is needed to examine this suggestion. Nevertheless, it raises the question of whether intensive in-home services would have been a better alternative for these children in order to prevent them from the risk of being traumatized by the out-of-home placement. The second issue involves the finding that the baseline level of psychosocial problems of residentially placed children was not (statistically) significantly higher compared to children in the family-oriented settings. This finding is contradictory to literature data that suggest that children with a high level of psychosocial problems are often assigned to residential care, among other things, to meet their high treatment needs (Butler and McPherson 2007; De Swart et al. 2012; Doran and Berliner 2001). The finding can possibly be attributed to the specificity of the study population which only consisted of Dutch out-of-home placed children. In the Netherlands, nowadays family-style group care is often assigned to children who need a long-term placement in a family-oriented setting at the end of a long care history, indicating that family-style group care instead of residential care seems to be used as treatment of “last resort” (Leloux-Opmeer et al. 2017).

### Strengths and Limitations

The strengths of this study include first the comparison of the psychosocial development of children placed in the three types of out-of-home care investigated. We found no other studies that conduct such a triple comparison. Second, this study contributes to the knowledge on out-of-home placement, particularly because we have taken into account the severity of the psychosocial problems present at the time of admission, which provided a more accurate impression of the children’s psychosocial development. Finally, we conducted analyses at group level as well as at individual level, which turned out to be a valuable addition.

However, the study also has some limitations. First, some of the analyses could have suffered from limited statistical power, due to the relatively small sample size of children in family-style group care. This can hamper the ability to find statistically significant associations, so the results should be interpreted with some caution. However, to provide insight into the relevance of the relationships explored in the study, we have added effect sizes for all the statistical findings, which also provides an impression of the power of the study (Cohen 1992). Second, we cannot rule out that regression to the mean partly determined the study results. Random allocation of subjects to treatment conditions is considered to be a valid strategy to resolve this issue (Barnett et al. 2005). However, for ethical reasons, random allocation of children to each of the three types of out-of-home care was no option in this study. Third, it should be noticed that other variables (e.g., quality of care, the child’s learnability, causes of psychosocial problems) might have affected the outcome variables. However, the aim of this study was to assess and mutually compare the psychosocial development of children during the first year after admission to the three care modalities concerned, taking into account possible differences in the severity of the children’s psychosocial problems at the time of admission. Further research is needed to investigate the influences that child, family, care history and specific treatment variables at the micro level have on the psychosocial development of children in 24-h care facilities. Finally, it is conceivable that other outcome measures than the degree of psychosocial functioning are important in the comparison of the development of children in the three types of out-of-home care.
